# The Synthesis and Characterization of a Delivery System Based on Polymersomes and a Xanthone with Inhibitory Activity in Glioblastoma

**DOI:** 10.3390/life14010132

**Published:** 2024-01-17

**Authors:** Ana Alves, Ana Margarida Silva, Claúdia Nunes, Sara Cravo, Salette Reis, Madalena Pinto, Emília Sousa, Francisca Rodrigues, Domingos Ferreira, Paulo C. Costa, Marta Correia-da-Silva

**Affiliations:** 1 UCIBIO—Applied Molecular Biosciences Unit, MedTech-Laboratory of Pharmaceutical Technology, Faculty of Pharmacy, University of Porto, Rua Jorge Viterbo Ferreira, 228, 4050-313 Porto, Portugal; 2 Laboratory of Organic and Pharmaceutical Chemistry, Department of Chemical Sciences, Faculty of Pharmacy, University of Porto, Rua Jorge Viterbo Ferreira, 228, 4050-313 Porto, Portugal; 3 Associate Laboratory i4HB—Institute for Health and Bioeconomy, Faculty of Pharmacy, University of Porto, Rua Jorge Viterbo Ferreira, 228, 4050-313 Porto, Portugal; 4 REQUIMTE/LAQV—Associated Laboratory for Green Chemistry, ISEP, Polytechnique of Porto, Rua Dr. António Bernardino de Almeida, 431, 4200-072 Porto, Portugal; 5 REQUIMTE/LAQV—Associated Laboratory for Green Chemistry, Department of Chemical Sciences, Faculty of Pharmacy, University of Porto, Rua Jorge Viterbo Ferreira, 228, 4050-313 Porto, Portugal; 6 Interdisciplinary Center of Marine and Environmental Research (CIIMAR), University of Porto, Terminal dos Cruzeiros do Porto de Leixões, Avenida General Norton de Matos, s/n, 4450-208 Matosinhos, Portugal

**Keywords:** xanthone, synthesis, glioblastoma, nanotechnology, polymersomes

## Abstract

Glioblastoma (GBM) is the most common and deadly primary malignant brain tumor. Current therapies are insufficient, and survival for individuals diagnosed with GBM is limited to a few months. New GBM treatments are urgent. Polymeric nanoparticles (PNs) can increase the circulation time of a drug in the brain capillaries. Polymersomes (PMs) are PNs that have been described as having attractive characteristics, mainly due to their stability, prolonged circulation period, biodegradability, their ability to sustain the release of drugs, and the possibility of surface functionalization. In this work, a poly(ethylene glycol)-ε-caprolactone (PEG-PCL) copolymer was synthesized and PMs were prepared and loaded with an hydrolytic instable compound, previously synthesized by our research team, the 3,6-bis(2,3,4,6-tetra-O-acetyl-β-glucopyranosyl)xanthone (XGAc), with promising cytotoxicity on glioblastoma cells (U-373 MG) but also on healthy cerebral endothelial cells (hCMEC/D3). The prepared PMs were spherical particles with uniform morphology and similar sizes (mean diameter of 200 nm) and were stable in aqueous suspension. The encapsulation of XGAc in PMs (80% encapsulation efficacy) protected the healthy endothelial cells from the cytotoxic effects of this compound, while maintaining cytotoxicity for the glioblastoma cell line U-373 MG. Our studies also showed that the prepared PMs can efficiently release XGAc at intratumoral pHs.

## 1. Introduction

According to the World Health Organization (WHO), glioblastoma (GBM) is classified in four grades, with grade I being characterized by least proliferating lesions with a possible cure, and grade IV by cytotoxic malignant neoplasms, which quickly proliferate and invade the surrounding healthy tissue [1]. Normally, a patient’s quality of life is poor, since the peripheral tissues must undergo treatment; if they do not undergo treatment, the tumor reappears. Therefore, treatments cause many side effects to healthy tissues [1].

Current treatments are still inadequate, and the prognosis after diagnosis tends to be poor, due to some tumor-related characteristics (such as being highly invasive, non-localized, and diffuse) and the difficulty to act locally [2,3]. Conventional surgical methods or radiotherapy alone cannot eliminate the tumor, and the relapse is, in most of the cases, inevitable [4]. The efficiency of current therapies is mainly compromised by the glioma cells’ complex composition, diffuse invasiveness, and blood–brain barrier (BBB) selectivity that inhibits drugs from infiltrating into the brain tumor mass.

The initial stage of the therapeutic process entails surgical intervention, which is subsequently supplemented by radiation therapy and a combined treatment regimen involving temozolomide (TMZ). This drug is the standard care in chemotherapy for GBM [5], and reached “blockbuster” status in 2010 [6]. Nevertheless, chemotherapy is difficult, due to the BBB efficacy and the heterogeneity of brain cancer [7].

A promising synthetic xanthone, the 3,6-bis(2,3,4,6-tetra-O-acetyl-β-glucopyranosyl)xanthone (XGAc), with inhibitory effects on the growth of GBM cell lines, was synthesized by our group [8]. XGAc has recently exhibited antitumor effects against triple-negative breast cancer (TNBC), ovarian cancer, and pancreatic ductal adenocarcinoma (PDAC) cells, both when administered alone and in conjunction with the poly(ADP-ribose) polymerase inhibitor (PARPi) Olaparib [9]. Due to its acetylated state, this xanthonoside demonstrates limited solubility in water and may undergo rapid hydrolysis by esterases. Consequently, encapsulating it within nanosystems presents a promising strategy for overcoming these constraints. Even though XGAc was successfully incorporated in liposomes, maintaining the inhibitory activity against GBM cell lines, these formulations have some major disadvantages, such as chemical and physical instability [10]. On the other hand, proliposomes, despite having successfully incorporated XGAc, showed some toxicity, perhaps due to the presence of mannitol in the formulation, which can provoke osmotic effects.

The aim of this study was to develop polymeric nanoparticles (PNs) for the drug delivery of this promising compound. PNs are a promising approach in cancer therapy due to their favorable characteristics that comprise wide structure variety and comparatively uncomplicated elaboration and design [11]. PNs can enhance circulation time in the brain capillaries and can take advantage of transcytosis pathways using different surface strategies. Furthermore, nanoparticles have the ability to escape the P-gp efflux pumps, owing to the presence of specific ligands engineered onto the particle surface [12]. Several PNs tend to show better results when compared with regular administration [13]. The most dominant classifications of FDA-approved medicines since 2016 were polymeric, accounting for 29%; liposomes, constituting 22%; and other lipid-based medicines, which comprised 21%.

A new generation of PNs, the polymersomes (PMs), were developed by Hammer and Discher, who were the firsts to describe the physical properties of these polymeric structures using poly (ethylene oxide)-block-poly (ethylene) diblock copolymers (PEO-b-PEE) that assemble themselves in aqueous environments [14]. Sequences of polymers are called blocks, and they can be diblock (A-B) or triblock (A-B-A or A-B-C) copolymers, depending on the number of polymer chains, being designated as versatile structures that can encapsulate hydrophilic or hydrophobic drugs [15]. There are a large variety of amphiphilic block copolymers, with different molecular weights being used to create smart or prolonged release formulations. The hydrophobic part, such as poly(ethylene) (PE), poly(butadiene) (PBT), poly(ethylene) (PBD), poly(dimethylsiloxane) (PDMS) and poly(styrene) (PS), poly(lactide) (PLA), poly(ε-caprolactone) (PCL), and poly(trimethylene carbonate) (PTMC), are biodegradable; the hydrophilic part, such as poly(acrylic acid) (PAA), poly(L-glutamic acid) (PGA), and poly(ethylene glycol) (PEG), are biocompatible. The molecular weight of the block copolymers is higher than phospholipids [16], providing large colloidal stability against mechanical shear and osmotic pressure in the bloodstream when compared to liposomes. The high molecular weight of PMs can expressively expand their membrane properties, such as thickness, permeability and robustness [17]. The mechanical stability of the PMs could be explained by their membrane thickness (5–20 nm), as they can be thicker than the liposome ones (3–5 nm). PMs have better physicochemical properties than liposomes, prolonged circulation time (PEG-coated liposomes have shown blood circulation time 2 to 3 days after intravenous injection [18,19]), and sustained drug release [20]. PMs already demonstrated low in vivo toxicity [21]. Doxorubicin and paclitaxel were loaded within PMs composed of poly(ethylene glycol)-poly(ε-caprolactone) PEG-PCL and poly(ethylene glycol)-polylactic acid PEG-PLA copolymers [22,23]. These PMs delivered these drugs to a tumor implanted in mice, and a 50% size reduction was reported five days after the drug was injected. In addition, PMs can be used as carriers for the delivery of genes, such as DNA or RNA.

In this study, PEG2000-PCL and PEG5000-PCL copolymers were synthesized and used to obtain different formulations of PMs, which were fully characterized. PEG was selected due to the resistance that provides in the superficial layers to the adsorption of proteins in the blood. PCL can exhibit a hydrolysis-triggered controlled release, which allows the release of drugs in response to specific conditions, and it can be easily functionalized, allowing for the development of targeted drug delivery systems [24,25]. The in vitro anti-growth activity of the PMs with XGAc and the release of XGAc from the PMs were also evaluated in this work.

## 2. Materials and Methods

### 2.1. General Information

Methoxy PEG 2000, Methoxy PEG 5000, ε-caprolactone, and Sn(oct)_2_ were acquired from Sigma-Aldrich Co. (Sintra, Portugal), and methanol (HPLC grade) from VWR chemicals. 1H and 13C-NMR spectra analysis was conducted at the Department of Chemistry at the University of Aveiro, Portugal., using a Bruker Avance 300 instrument (1H: 300.13 MHz; 13C: 75.47). 13C-NMR assignments were made through bidimensional HSQC and HMBC experiments (long-range C, H-coupling constants were optimized to 7 and 1 Hz). Chemical shifts are expressed in ppm values relative to tetramethylsilane (TMS) as an internal reference, and coupling constants are reported in hertz (Hz). Cells’ reagents were purchased from Gibco (Invitrogen Corporation, Life Technologies, Renfrew, UK). Immortalized human brain capillary endothelial cells (hCMEC/D3 cell line) were kindly donated by Dr. PO Couraud (INSERM, Paris, France). Human astrocytoma U87-MG cell line was purchased from American Type Culture Collection (ATCC). For hCMEC/D3 and U-87 MG, respectively, passages 48–49 and 30–35 were used.

### 2.2. The Synthesis and Stability Studies of XGAc

XGAc was synthesized according to the previously reported method [8]. Stability studies of XGAc were performed by LC-UV quantification method. A calibration curve for XGAc was prepared in ethanol at the concentrations of 0.5, 1, 2, 5, 10, 20, 40 µM in triplicate.

LC-UV method was carried out in a High Performance Liquid Chromatography (HPLC) system Dionex UltiMate™ 3000 (Thermo Fisher Scientific, Bremen, Germany), equipped with a quaternary pump and a quaternary Variable Wavelength Detector. Chromeleon 7.0 software was used for data acquisition. The chromatographic conditions included a commercially available Fortis 5 µm UniverSil C18 (250 × 4.6 mm) column from Fortis Technologies Ltd. (Neston, Cheshire, UK). The optimized mobile phase consisted of water:acetonitrile:acetic acid (35:65:0.2, *v*:*v*:*v*) following a isocratic flow of 1.0 mL/min for 15 min. The temperature of the column was set at room temperature. The volume of injection was 20 μL, and the detection was performed at 237 nm. XGAc retention time was 5.9 min.

The stability study was conducted at −20 °C, 4 °C, 25 °C, and 37 °C in three different buffer solutions prepared at pH 4, pH 7.4, and pH 9. A stock solution of XGAc was prepared at 1 mM in DMSO, and 10 µL of this stock solution was diluted to 1 mL of the different buffers before thermal incubation. A 100 µL aliquot was removed immediately before LC-UV analysis at 0, 1, 2, 24, 48, 120, and 360 h. The assays were conducted in triplicate.

### 2.3. The Synthesis and Preparation of Polymersomes

Ring-opening polymerization was employed to synthesize the PEG-PCL diblock copolymer, facilitated by microwave assistance. In summary, the reaction was executed by subjecting it to microwave irradiation: firstly, 2.5 g of PEG2000 and methoxyPEG5000 was dried at 120 °C and 1000 W for 10 min; then, 6.55 g ε-caprolactone (PCL) and 10 μL Sn(oct)_2_ were added to the dried methoxyPEG; the reaction continued at 130 °C for 25 min, and was stirred at 30 rpm and 1000 W irradiation. For purification, the synthesized copolymer was dissolved in chloroform and then precipitated by adding an adequate amount of diethyl ether. This experiment was performed three times, and then the precipitate was freeze-dried to eliminate any remaining water. Afterward, the copolymer was stored at a temperature of −20 °C. The NMR spectrum of PEG-PCL diblock was obtained at room temperature in CDCl_3_.

The PMs structures were prepared through the film rehydration method. A total of 20 mg of the copolymer and 5 mg of XGAc were dissolved in 2 mL of dichloromethane. The solvent was evaporated under vacuum. The thin polymer film was hydrated with 2 mL distilled water at 60 °C and stirred overnight (1250 rpm continuous stirring). The polymer dispersion was sonicated for 30 min at 25 °C, followed by extrusion 20 times through a homogenizer (FPG12800 Pressure Cell Homogenizer, Unit 5 New Horizon Business Center Barrows Road Harlow, Essex, CM19 5FN, Stansted, UK).

### 2.4. The Characterization of Polymersomes

#### 2.4.1. Particle Size and Polydispersion Index

The sample was prepared by adding 40 μL of PMs sample in 1960 μL of purified water and was analyzed using Dynamic Light Scattering (DLS) with a ZetaPALS apparatus (Brookhaven Instruments Corporation, Holtsville, NY, USA). The collected data, mean diameter, and polydispersion index (PDI), was obtained by PALS Particle Sizing Software (Version 5, Brookhaven Instruments Corporation, Holtsville, NY, USA) and was expressed as mean ± standard deviation throughout the work.

#### 2.4.2. Thermal Behavior

The investigation into the excipient–excipient, and XGAc–excipient compatibility studies was made with Differential Scanning Calorimetry (DSC) with a DSC 200 F3 Maia (Netzsh–Gerätebau GmbH, Selb, Germany). PMs’ formulations and the isolated compounds (XGAc and excipients) were weighed in DSC aluminum pans and the experiment was conducted within a controlled nitrogen atmosphere, with a flow rate of 40 mL/min. The temperature range spanned from −40 to 340 °C, with a heating rate of 10 °C/min. An empty aluminum pan was used as a reference. The results were analyzed using Proteus Analysis software (Version 6.1, Netzsh-Gerätebau GmbH, Germany). The DSC apparatus was calibrated (temperature and sensitivity) using the following standards: Hg (m.p. −38.8 °C), In (m.p. 156.6 °C), Sn (m.p. 231.9 °C), Bi (m.p. 271.4 °C), Zn (m.p. 419.5 °C), and CsCl (m.p. 476.0 °C).

#### 2.4.3. Negative-Staining Transmission Electronic Microscopy

For negative staining transmission electron microscopy, 10 µL of samples were mounted on Formvar/carbon film-coated mesh nickel grids (Electron Microscopy Sciences, Hatfield, PA, USA) and left standing for 2 min. The liquid in excess was removed with filter paper, and 10 µL of 1% uranyl acetate was added on to the grids and left standing for 10 s, after which liquid in excess was removed with filter paper. Visualization was carried out on a JEOL JEM 1400 TEM at 120 kV (Tokyo, Japan). Images were digitally recorded using a CCD digital camera Orious 1100W (Tokyo, Japan). The transmission electronic microscopy was performed at the HEMS core facility at i3S, University of Porto, Portugal.

#### 2.4.4. Entrapment Efficiency

The obtained formulation was centrifuged (4500 rpm for 15 min) (Model 5804, Eppendorf, Hauppauge, NY, USA), and the supernatant was filtered (0.45 μm PTFE filter, OlimPeak^®^, Teknokroma, Barcelona, Spain) to obtain the XGAc free amount sample. The obtained samples were then evaluated by HPLC (Dionex UltiMate™ 3000 from Thermo Fisher Scientific, Bremen, Germany) equipped with a quaternary pump and a quaternary Variable Wavelength Detector. Chromeleon 7.2 software was used for data acquisition.

The chromatographic conditions included a commercially available Acclaim^TM^ 120 C18 (100 × 4.6 mm) column with particle size 5 µm, from Thermo Fisher Scientific (Bremen, Germany). The optimized mobile phase consisted of water:methanol (25:75, *v*:*v*), following a isocratic flow of 1.0 mL/min for 10 min, and the temperature of the column was set at room temperature. The volume of injection was 10 μL, and the detection was performed at 265 nm. XGAc retention time was 3.3 min.

A calibration curve for XGAc was prepared in methanol at concentrations of 28, 30, 36, 52, and 62 μg/mL in triplicate, and the drug concentration in supernatant was obtained by interpolation on the calibration curve. The theoretical concentration of XGAc was calculated by considering the amount of XGAc placed during the production of the PMs and the dilutions performed throughout the procedure, and the encapsulation efficiency (EE) can be calculated using Equation (1):

EE (%) = TA − FA/TA(1)

where, TA (total amount) was the initial concentration of the XGAc in the formulation (μg/mL) and FA (free amount) was the final free concentration of the XGAc in the supernatant (μg/mL).

#### 2.4.5. Stability of PMs

The stability investigation was conducted on the PMs, both with and without XGAc. The samples were intermittently assessed for the mean diameter and PDI at time points 0 and 1, 7, and 15 days post-manufacturing, while the formulations were preserved at a temperature of 4 °C.

#### 2.4.6. MTT Cell Viability Assay

Cells were cultivated as reported by Mendes et al. [26]. The human glioblastoma astrocytoma derived from a malignant tumor (U-373 MG) was obtained from Sigma-Aldrich. Immortalized human brain capillary endothelial cells (hCMEC/D3 cell line) were kindly donated by Dr. PO Couraud (INSERM, France). Human astrocytoma U87-MG cell line was purchased from American Type Culture Collection (ATCC) and cell reagents were provided by Life Technologies, S.A. (Madrid, Spain) and Biowest (Nuaillé, France). Passages 53–56, 48–49 and 16–17, respectively.

For the 3-(4,5-dimethylthiazol-2-yl)-2,5-diphenyltetrazolium bromide (MTT) assay, cells were seeded in 96-well plates (25 × 10^3^ cells/mL) and exposed to different concentrations (0.1, 1, 10, 100 μM) of PEG2000-PCL and PEG5000-PCL without and with XGAc for 24 h. After extracting the formulations from each well, the cells underwent a thorough washing with HBSS. The quantification of viable cells was conducted by introducing MTT reagent and allowing for an incubation period of 3 h at a temperature of 37 °C. To dissolve the crystals, DMSO was employed. Triton X-100 1% (*w*/*v*) (0.00 ± 0.56%) and culture medium (105.00 ± 11.36%) were used as negative and positive controls, respectively. The absorbance was read at 590 nm with background subtraction at 630 nm. Results were expressed as percentages of cell viability.

#### 2.4.7. The In Vitro Release of XGAc

In vitro acetylated xanthonoside release studies were performed using a cellulose dialysis bag diffusion technique (Spectra/Por 3 dialysis tubing 3.5 kDa) filled with 1 mL of the sample PEG2000-PCL-XGAc, and PEG5000-PCL-XGAc suspended in isotonic phosphate-buffered solution (PBS), pH 6.2. The dialysis membranes were placed in 80 mL of PBS, pH 6.2 under magnetic stirring at 150 rpm and maintained at 37 °C. At fixed time intervals (0, 1, 2, 3, 4, 5, 6, 7, 8, 24, 48, 72, and 96 h), 1 mL of the PBS solutions were withdrawn and the solution obtained was analyzed by HPLC to determine the concentration of the XGAc, as previously described (Section 2.4.4). The studies were performed in triplicate and the cumulative percentage of the released compound was determined by calculating the mean value.

The release profiles were compared and fitted to the following kinetic models: Higuchi, zero-order, and Korsmeyer–Peppas (Equations (2)–(4), respectively):

Q_t_ = Q_0_ + K_Hh_t^1/2^ (2)

Q_t_ = Q_0_ + K_0_t (3)

Q_t_ = Q_0_ + K_KP_t^n^ (4)

where Q_t_ is the amount of XGAc released in time t, Q_0_ is the initial amount of XGAc released, K_H_ is the Higuchi dissolution constant, K_0_ is the zero-order release constant, K_KP_ is the Korsmeyer–Peppas release constant, and n is the release exponent [27]. Model fitting was performed in MS Excel version 16.80, using the Solver add-in. Adjusted R-squared ${(R}_{adj}^{2})$ should be used when comparing kinetic models with different numbers of parameters [28], and can be calculated from Equation (5):

(5) $$R_{adj}^{2}=1–\frac{\left(1-R^{2})(q-1\right)}{q-p-1}$$

where q is the number of experimental data points and p is the number of parameters.

### 2.5. Statistical Analysis

The results of cell viability, mean diameter, and EE were statistically analyzed using ANOVA, after confirming normality and homoscedasticity (Shapiro–Wilk and Levene tests). Differences between groups were compared with Tukey’s HSD and different letters in the same sample represented significant differences between different concentrations. Significance was set at *p* < 0.05. All the statistical analyses were performed with IBM SPSS Statistics for Windows (Version 28.0., IBM Co., Armonk, NY, USA). All the results are shown as mean ± standard deviation of three batches of the same formulation (*n* = 3).

## 3. Results

### 3.1. The Stability Studies of Synthetic XGAc

**Figure 1:**
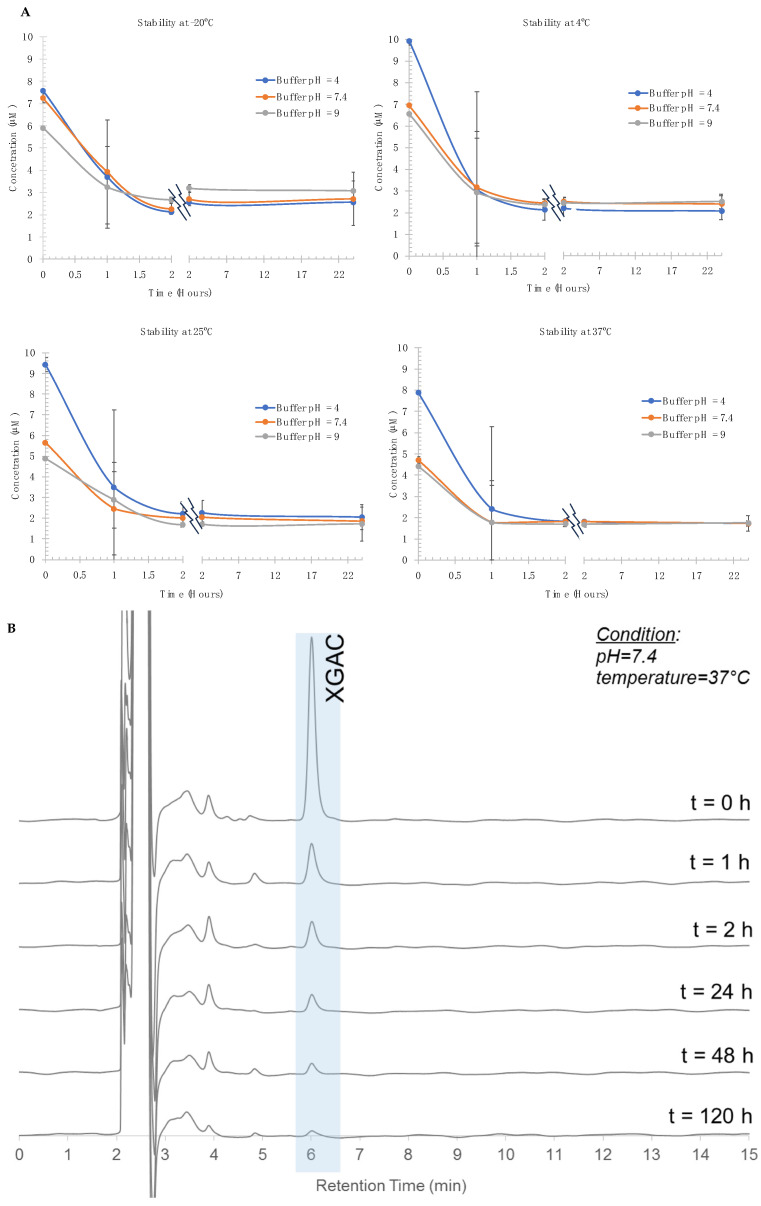
(**A**) Stability of XGAc at −20, 4, 25, and 37 °C versus time; values are expressed as mean ± standard deviation (*n* = 2); (**B**) Examples of an XGAc chromatogram, pH = 7.4 at 37 °C at different times.

Data obtained in the stability studies were fitted to the least squares linear regression (y = 3762.1x − 4938.2). The determination coefficient (R^2^) was 0.9963, showing a good linearity in the XGAc-tested range. The results show that, independently of the pH and temperature, XGAc is unstable and undergoes a rapid degradation process in the first 24 h of incubation (Figure 1). The most stable conditions are at −20 °C, but the fact that to collect the aliquots to do the LC-UV analysis, we need to defrost the buffers solutions, considering the stability profile of the incubations at room and 37 °C temperature, and the defrost process being a thermal one, it may trigger the degradation process in the −20 °C assays.

### 3.2. The Synthesis and Characterization of the PEG-PCL Diblock Copolymer

**Figure 2:**
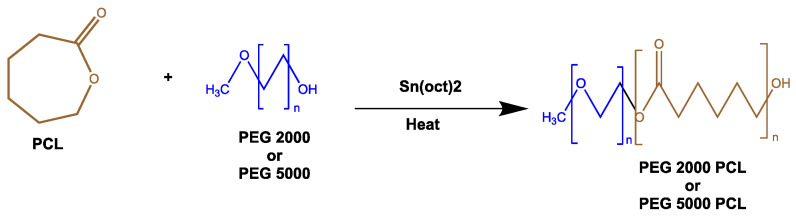
Synthesis of PEG2000-PCL and PEG5000-PCL by ring-opening polymerization.

The amphiphilic PEG2000-PCL and PEG5000-PCL diblock copolymers were obtained by the ring-opening polymerization (ROP) method (Figure 2). The most commonly used catalyst is stannous octoate (Sn(Oct)_2_) [29,30]. The macroinitiator, methoxy polyethylene glycol (methoxyPEG), must be dried in a microwave [31], and a variety of hydrophobic building blocks, such as ε-caprolactone (PCL), have been used for the PMs formation [32,33,34].

For both copolymers, the synthesis by microwave irradiation was simple, rapid, and highly efficient.

The ring-opening polymerization (ROP) of ε-caprolactone occurs when Sn(Oct)_2_ is added to the ε-caprolactone monomer, causing the polymer to form [35]. The initiation step begins with the coordination of the carbonyl oxygen of the ε-caprolactone with the tin atom. PEG serves as a macromolecular initiator for the polymerization of ε-caprolactone. The polymerization process and chain growth continues when the Sn(Oct)_2_ primer attacks the carbonyl oxygen of another ε-caprolactone molecule.

Also, through Equation (6), it is possible to observe that the reaction happened successfully, with PEG2000-PCL presenting 45 monomers of PEG2000 and 83 monomers of PCL, while PEG5000-PCL presented 114 monomers of PEG5000 and 108 of PCL. This shows that the polymerization of ε-caprolactone occurred.

NM = (δCL)/[(Protons of CL/Protons of EG) × δ PEG] × Mw PCL (6)

δCL = sum of the signals of the CL; Mw PCL = molecular weight of PCL; protons of CL = number of protons of CL; protons of EG = number of protons of PEG.

**Figure 3:**
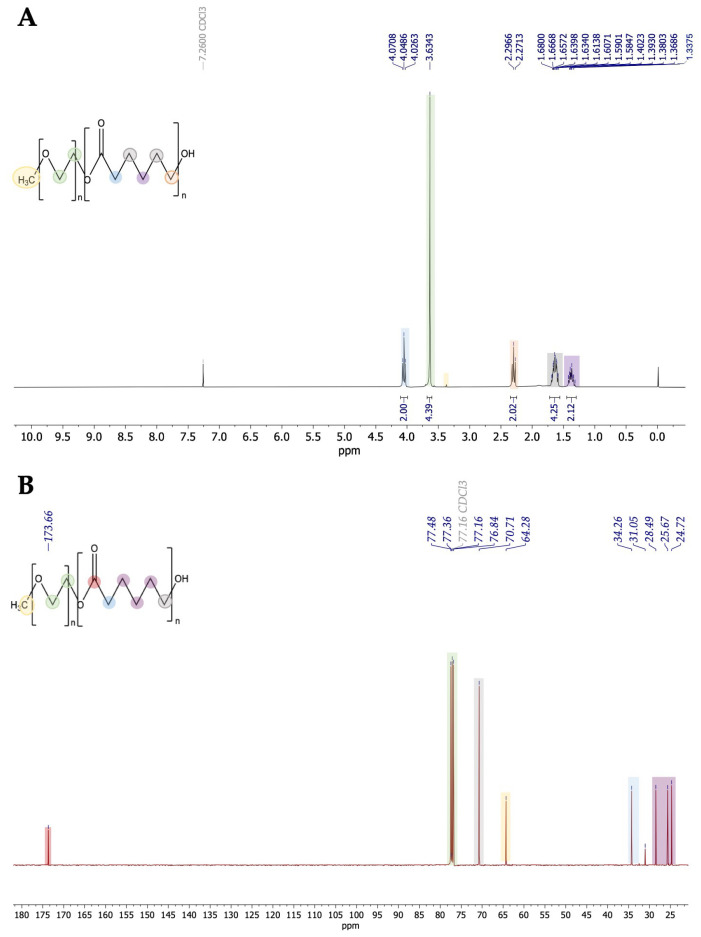
(**A**) ^1^H NMR spectrum (CDCl_3_) of PEG5000-PCL copolymers; (**B**) ^13^C NMR spectrum (CDCl_3_) of PEG5000-PCL copolymers.

**Figure 4:**
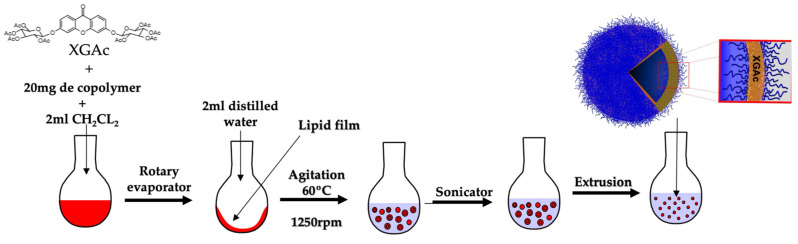
Preparation and assembly of the PMs by film rehydration method.

Figure 2 shows the ^1^H NMR spectrum of PEG5000-PCL diblock. The characteristic OCH_2_CH_2_O of the PEG block was assigned to the chemical shift 3.6 ppm (green, Figure 3). The chemical shift at 4.1 ppm was assigned to CH_2_ alpha carbonyl of the PCL block (blue, Figure 3). The signals of the other CH_2_ protons of the PCL block appeared at 2.3 and 1.6 ppm as multiplets. Figure 4 shows the ^13^C NMR spectrum of PEG5000-PCL diblock. Regarding PEG segment, the aliphatic carbons were detected at 77.4 and 77.3 ppm, and the methylene carbon at 64.2 ppm. Regarding the PCL segment, the carbonyl of the ester -COO- was assigned to the chemical shift 173.6 ppm, the carbon of the hydroxyl to 76.8 ppm, the CH_2_ alpha carbonyl to 34.2 ppm, and the aliphatic carbons to 28.4, 25.6, and 24.7 ppm.

### 3.3. The Preparation and Characterization of Polymersomes

By the film rehydration method (Figure 4), the PMs successfully self-assembled in water. The lipophilic nature of XGAc, insoluble in water, allowed us to predict that this drug was incorporated in the hydrophobic part of PMs. The mean diameter and PDI of PMs can be seen in Table 1. The capacity of particles to successfully travel to the interstitial space through tumor vessel walls depend on the ratio of particle size/opening size. Overall, the decrease in the particle size improves the transport through tumor vessel walls.

The presence of XGAc in the PEG5000-PCL formulation did not affect the size (164.23 nm, *p* > 0.05), in contrast to the PEG2000-PCL-XGAc formulation (276.33 nm, *p* > 0.05). Empty formulations of PEG2000-PCL particles were larger than PEG5000-PCL particles, 128.56 nm and 112.13 nm, respectively. As stated before, smaller size formulations can increase the drug’s effectiveness. Tumor tissues, fenestrations and the deterioration of blood vessels are common as a result of the rapid and irregular angiogenesis. These are open doors for smaller particles; therefore, the smaller the PMs, the greater the possibility of leaking into the tumor interstitial fluid, leading to the accumulation and eventually the destruction of tumor cells.

The PDI value of 0.1 to 0.3 represents nearly monodisperse preparation, whereas PDI  > 0.4 suggests a broad distribution of macromolecular sizes in solution, and non-monomodal distribution methods should be considered for data analysis [36]. For all formulations studied, the PDI was around 0.3, which is usually indicated as a limit for monodisperse preparations.

#### 3.3.1. Thermal Behavior

**Figure 5:**
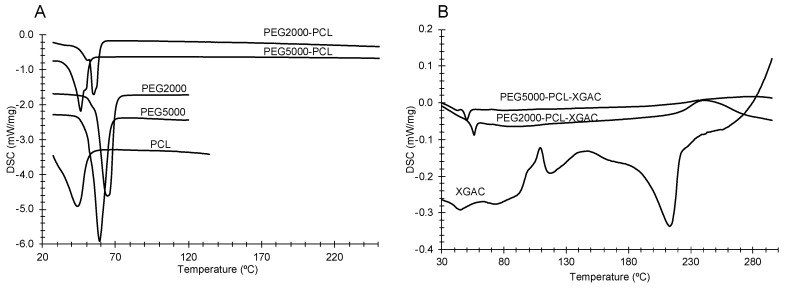
Thermograms of the components of the empty diblock copolymer and their constituents, (**A**) and thermograms of pure XGAc and PMs with XGAc (**B**).

DSC thermograms of the PMs’ formulation components are shown in Figure 5A. For the PEG2000 and PEG5000, the onset temperatures were 60 °C and 52 °C, respectively. For the PCL, the onset was −11 °C. For the mixtures, PEG2000-PCL and PEG5000-PCL, the onset temperatures were 40 °C and 31 °C, respectively, showing that a small amount of PCL influenced the crystallization behavior of PEG. Nevertheless, the varied onset indicated that the crystalline structure for both PCL and PEG were altered. The pure XGAc and the PM formulations with XGAc are shown in Figure 5B. XGAc has an onset at 195 °C and the PEG2000-PCL-XGAc and PEG5000-PCL-XGAc PMs presented only a peak with an onset at 48 °C and 53 °C, respectively.

The inclusion of XGAc and the formation of PMs provoked a relevant change in the PEG2000-PCL and PEG5000-PCL crystalline forms, shown by the variation in enthalpy values from 112 J/g to 1.02 J/g and 102 J/g to 3.20 J/g, respectively. These results suggest that XGAc is molecularly dispersed in the formulations, as can be seen by the absence of its peak.

#### 3.3.2. A Negative-Staining Transmission Electron Microscopic Study

**Figure 6:**
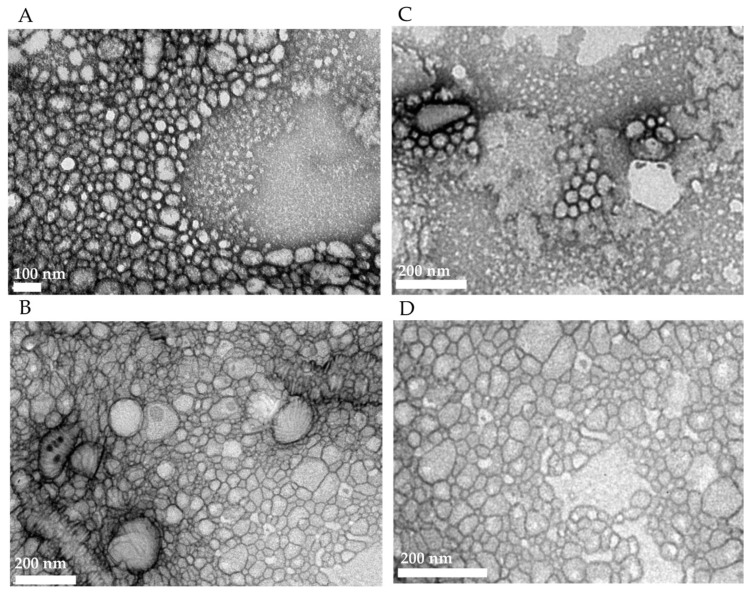
TEM images of (**A**) PEG5000-PCL formed by film rehydration; (**B**) PEG5000-PCL-XGAc formed by film rehydration; (**C**) PEG2000-PCL formed by film rehydration; (**D**) PEG2000-PCL-XGAc formed by film rehydration.

The images of the particles were obtained using a transmission electron microscopic (TEM) technique, aiming to evaluate their morphology. Figure 6A,B show the morphology of the particles of PEG5000-PCL and PEG5000-PCL-XGAc, respectively, obtained by film rehydration. The particles had a spherical shape and the presence of XGAc in PMs particles did not alter their morphology. Both types of particles showed small diameters, but it is possible to observe larger diameter particles.

Figure 6C,D show the PEG2000-PCL and PEG2000-PCL-XGAc, respectively, obtained by the film rehydration method. It can be observed that the spherical shape of PEG2000-PCL was not modified with the presence of XGAc.

#### 3.3.3. Entrapment Efficiency

The XGAc UV spectrum was performed, varying the wavelength from 200 to 400 nm. One of the XGAc absorption bands occurred at 265 nm wavelength, which was selected to calculate the EE by HPLC. Data were fitted to the least squares linear regression (y = 0.1189x − 0.0777). The R^2^ was 0.9996, which exhibits a good linearity in the tested XGAc range.

The EE of the compound in diblock copolymers was determined by the indirect measurement of the XGAc that was encapsulated in the formulation by HPLC. Both formulations, PEG5000-PCL and PEG2000-PCL, had high drug EE, respectively, 87.3% and 71.2%.

#### 3.3.4. The Stability of PMs

**Figure 7:**
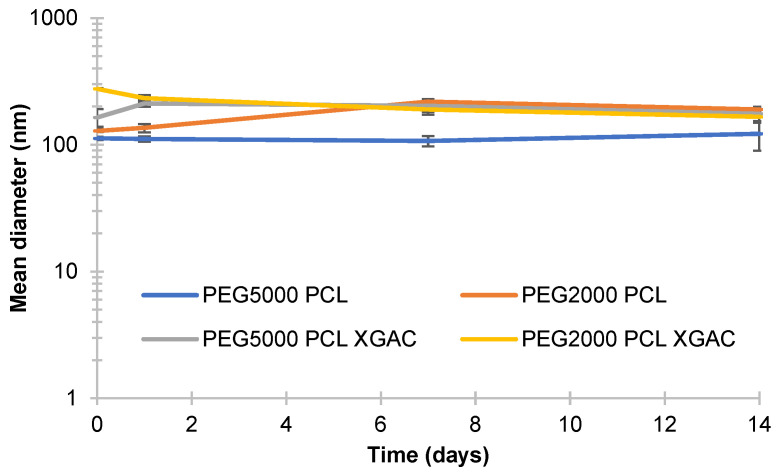
Effective mean diameter of diblock copolymers PMs, with and without XGAc, from the day of production to 1, 7, and 14 days after (*n* = 3).

Polymersomes were stored at 4 °C to understand their suitability for long-term storage in an aqueous suspension. The formulations showed no apparent change after a storage period of 1, 7, and 14 days, indicating the probable stability of the prepared diblock copolymers PMs (Figure 7).

PEG5000-PCL particles’ sizes without XGAc did not present significant differences over time (*p* = 0.444). In contrast, one day after preparation, the PEG5000-PCL with XGAc increased the mean diameter from 164.23 nm to 211.77 nm (*p* = 0.049), 7 days after showed a mean diameter of 202.80 (*p* = 0.562), and after 14 days, the particles with and without XGAc presented mean diameters of 175.33 nm (*p* = 0.090) and 122.06 nm (*p* = 0.482), respectively.

Regarding the PEG2000-PCL particles without XGAc, one day after preparation, the formulations did not present significant differences (*p* = 0.323), whereas PEG2000-PCL with XGAc showed a mean diameter of 232.43 nm (*p* = 0.0254). PEG2000-PCL without XGAc, after 7 days of the production, increased the mean diameter from 135.83 nm to 218.73 nm (*p* = 0.0006), while PEG2000-PCL with XGAc decreased the mean diameter from 232.43 nm to 189.27 nm (*p* = 0.0254). After 14 days of production, the particles with and without XGAc decreased in mean diameters to 189.60 nm (*p* = 0.020) and 165.87 nm (*p* = 0.155).

As PEG2000-PCL PMs showed some significant changes over time, while the PEG5000-PCL PMs demonstrated good stability up to 14 days when stored at 4 °C, it seems that PEG5000-PCL PMs are more stable than the PEG2000-PCL PMs.

Previous studies on the stability of PMs have also shown that PMs stored at 4 °C demonstrate excellent stability for up to 30 days when stored as an aqueous suspension [37].

#### 3.3.5. Cell Viability Effects

The viability of cerebral endothelial cell lines (hCMEC/D3) and glioblastoma (U-87 MG and U-373 MG) after exposure to PMs formulations PEG2000-PCL and PEG5000-PCL, with and without XGAc, was evaluated by MTT.

**Figure 8:**
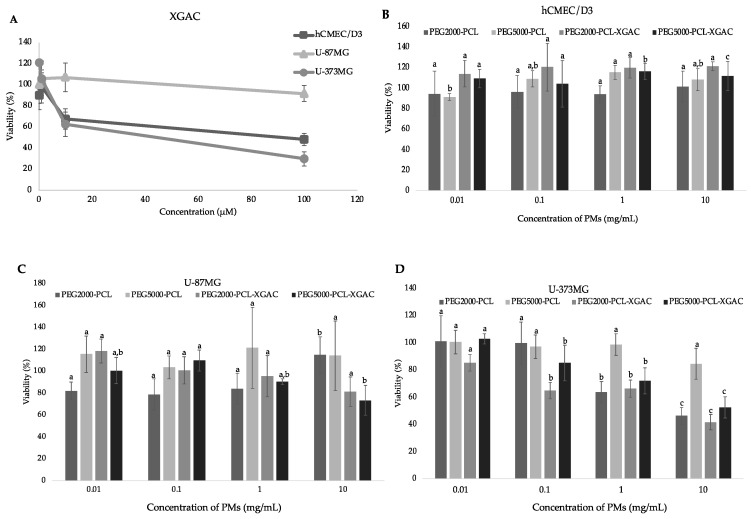
Viability of hCMEC/D3, U-87MG, and U-373MG cell lines (MTT assay) after exposure to at different concentrations of XGAc (**A**); effects of PMs with and without XGAc on the viability (MTT assay) of hCMEC/D3, (**B**) U-87 MG, (**C**) and U-373 MG (**D**) cell lines. Values are expressed as mean ± standard deviation (*n* = 3). Different letters (a,b,c) in the same sample represent significant differences (*p* < 0.05) between different concentrations, according to Tukey’s HSD test.

The effect of the free compound in the hCMEC/D3 cell line was evaluated for the first time, showing cytotoxicity to this cell line growth from 67.92% to 48.25% at 10 μM and 100 μM, respectively (GI_50_ = 18.01 μM). The cell growth of U-87MG and U-373MG cell lines in the presence of XGAc (Figure 8A) was affected similarly to that previous described [8].

The PMs’ formulations of PEG2000-PCL and PEG5000-PCL did not affect brain endothelial cell lines’ viability at any concentration tested (Figure 8B). The encapsulation of XGAc in these PMs successfully protected brain endothelial cell lines from the cytotoxicity of this compound.

At all concentrations tested, PMs with and without XGAc did not affect the cell viability of glioblastoma cells U-87 MG (Figure 8C).

In contrast, the MTT assay in U-373MG glioblastoma cells (Figure 8D) showed that empty PEG2000-PCL PMs and PEG2000-PCL-XGAc, decreased cell viability to less than 50% at 10 mg/mL. At all concentrations tested, empty PEG5000-PCL PMs did not affect U-373 MG viability, while PEG5000-PCL-XGAc PMs decreased the cell growth of this glioblastoma cell line to 52.57% at 10 mg/mL.

Overall, while the PMs with and without XGAc showed no toxicity for the brain endothelial cell lines, hCMEC/D3, the prepared formulations showed cytotoxicity at concentrations above 1 mg/mL for the glioblastoma cell line U-373 MG.

#### 3.3.6. The In Vitro Release of XGAc

**Figure 9:**
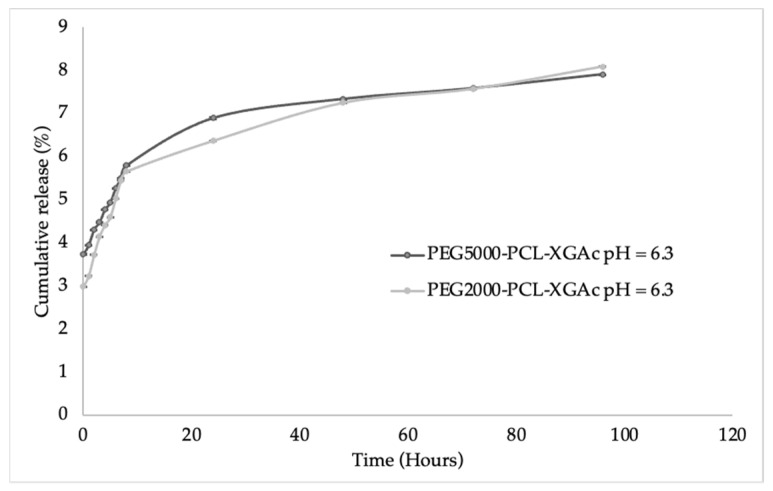
In vitro release profiles of acetylated xanthonoside (XGAc) from PEG2000-PCL and PEG5000-PCL PMs, at pH 6.3 at 37 °C.

In vitro release studies of XGAc were carried out in an aqueous buffer solution at pH 6.2 (acidic, to mimic the intratumoral pH) from PMs formulations over time for up to 96 h. Figure 9 shows the release curves of XGAc from the PEG2000-PCL-XGAc and PEG5000-PCL-XGAc PMs. The XGAc release amounts over time were 2.99% to 8.09% from PEG2000-PCL PMs and 3.73% to 7.92% from PEG5000-PCL PMs, which showed a sustained release pattern for both formulations, indicating that these PMs’ formulation method is suitable for a nanostructured sustained drug release system [38].

The initial release of XGAc from PEG5000-PCL was 3.73% to 5.80% in the first 8 h and increased after 24 h from 5.80% to 6.90%; at 72 h, there was still release from 6.90% to 7.59%, and finally, at the last time point, there was a release of XGAc of 7.92%.

Regarding the release from PEG2000-PCL PMs, the XGAc release curve increases from 2.99% to 5.66% in the first 8 h, after 48 h it increases from 5.66% to 7.25%, and at 96 h it was released up to 8.09%.

In general, the release studies of XGAc in PEG-PCL PMs showed that XGAc can be efficiently released from the PMs at intratumoral pHs.

The results for the kinetic modeling of the XGAc release profiles from the PEG-PCL are presented in Table 2. The release data of PEG2000-PCL-XGAc and PEG5000-PCL-XGAc fitted best in Korsmeyer-Peppas model ($R_{adj}^{2}$ > 0.938). The $R_{adj}^{2}$ values obtained for the Higuchi model were 0.909 and 0.914, respectively. Finally, the $R_{adj}^{2}$ values obtained for zero order model were 0.735 and 0.738, respectively. The values of n were 0.344 and 0.361 for PEG2000-PCL-XGAc and PEG5000-PCL-XGAc, respectively, showing a similar release mechanism. In polydisperse microparticles, the Fickian diffusion mechanism can be distinguished by values of *n* < 0.43 [39]. Ritger e Peppas [40] mentioned that n values close to 0.3 are also possible for Fickian diffusion in spherical samples with high polydispersion.

According to the Korsmeyer-Peppas model and regarding the Q_0,_ can be observed in PEG2000-PCL-XGAc and PEG5000-PCL-XGAc formulations (4.277% and 4.716%, respectively). The values of K for this model were higher in the PEG2000-PCL-XGAc (1.1672), showing a higher release rate in comparison to PEG5000-PCL-XGAc (0.921). This higher release rate compensated for a lower burst effect for PEG2000-PCL-XGAc, so that both formulations reached a similar release plateau after 48 h.

## 4. Conclusions

Due to the presence of acetyl groups, the anti-GBM compound XGAc has poor water stability, and will suffer rapid hydrolysis that will lead to a rapid degradation in the first 24 h. In this work, PMs were developed to encapsulate this synthetic compound. Two copolymers, PEG5000-PCL and PEG2000-PCL, were successfully synthesized by ROP. PMs presented spherical particles with uniform morphology and similar size, showing a mean diameter of 200 nm, approximately. PEG5000-PCL PMs demonstrated excellent stability for up to 14 days when stored at 4 °C in an aqueous suspension, while PEG2000-PCL PMs showed some significant changes over time. DSC showed that XGAc was molecularly dispersed in the formulations. The encapsulation efficacy of XGAc was approximately 80%. In contrast to free XGAc, XGAc encapsulated in PMs did not show any cytotoxicity to brain endothelial cell lines, while maintaining cytotoxicity against U-373MG at concentrations higher than 1 mg/mL. Release studies of XGAc from the prepared PMs showed a sustained release of this anti-glioblastoma compound at intratumoral pHs. The mechanism of drug release can be explained by Fickian diffusion in spherical samples with high polydispersion.

Overall, it seems that these diblock PMs provided enough hydrophobicity to load hydrophobic compounds, such as XGAc, protecting this compound from hydrolysis and brain endothelial cell lines from the cytotoxic effects of this compound.

## Figures and Tables

**Table 1:** Effective diameter and polydispersion index for PMs particles of diblock copolymers prepared by film rehydration method (*n* = 3).

Formulations	Mean Effective Diameter (nm) ± sd	PDI
PEG2000-PCL	128.56 ± 1.56	0.369 ± 0.002
PEG2000-PCL-XGAc	276.33 ± 5.60	0.376 ± 0.005
PEG5000-PCL	112.13 ± 0.86	0.168 ± 0.009
PEG5000-PCL-XGAc	164.23 ± 4.93	0.310 ± 0.009

PEG = methoxy polyethylene glycol; PCL = ε-caprolactone; PDI = polydispersion index; sd = standard deviation.

**Table 2:** Estimated kinetic parameters of the different mathematical models fitted to the XGAc release from PEG2000-PCL-XGAc and PEG5000-PCL-XGAc PMs.

Kinetic Model	Parameters	PEG2000-PCL-XGAc	PEG5000-PCL-XGAc
Zero-order	$K_{0}$	0.047	0.041
	Q_0_	4.277	4.716
	$R_{adj}^{2}$	0.735	0.738
Higuchi	$K_{H}$	0.524	0.454
	Q_0_	3.401	3.958
	$R_{adj}^{2}$	0.909	0.914
Korsmeyer-Peppas	$K_{KP}$	1.167	0.921
	Q_0_	2.684	3.429
	$R_{adj}^{2}$	0.938	0.935
	n	0.344	0.361

## Data Availability

Data are contained within the article.
